# A prediction model based on artificial intelligence techniques for disintegration time and hardness of fast disintegrating tablets in pre-formulation tests

**DOI:** 10.1186/s12911-024-02485-4

**Published:** 2024-03-27

**Authors:** Mehri Momeni, Marziyeh Afkanpour, Saleh Rakhshani, Amin Mehrabian, Hamed Tabesh

**Affiliations:** 1 Department of Medical Informatics, Faculty of Medicine, Mashhad University of Medical Sciences, Mashhad, Iran; 2 Department of pharmaceutics, school of pharmacy, Mashhad University of Medical Sciences, Mashhad, Iran; 3 Nanotechnology Research Center, Pharmaceutical Technology Institute, Mashhad University of Medical Sciences, Mashhad, Iran

**Keywords:** Pharmaceutical formulation, Orally disintegrating tablets (ODTs), Machine learning, Deep learning, Drug design, Tablet disintegration time, Tablet hardness, Pharmaceutical innovation, Prediction model

## Abstract

**Background:**

The pharmaceutical industry is continually striving to innovate drug development and formulation processes. Orally disintegrating tablets (ODTs) have gained popularity due to their quick release and patient-friendly characteristics. The choice of excipients in tablet formulations plays a critical role in ensuring product quality, highlighting its importance in tablet creation. The traditional trial-and-error approach to this process is both expensive and time-intensive. To tackle these obstacles, we introduce a fresh approach leveraging machine learning and deep learning methods to automate and enhance pre-formulation drug design.

**Methods:**

We collected a comprehensive dataset of 1983 formulations, including excipient names, quantities, active ingredient details, and various physicochemical attributes. Our study focused on predicting two critical control test parameters: tablet disintegration time and hardness. We compared a range of models like deep learning, artificial neural networks, support vector machines, decision trees, multiple linear regression, and random forests.

**Results:**

A 12-layer deep neural network, as a form of deep learning, surpassed alternative techniques by achieving 73% accuracy for disintegration time and 99% for tablet hardness. This success underscores its efficacy in predicting complex pharmaceutical factors. Such an approach streamlines the drug formulation process, reducing iterations and material consumption.

**Conclusions:**

Our findings highlight the deep learning potential in pharmaceutical formulations, particularly for tablet hardness prediction. Future work should focus on enlarging the dataset to improve model effectiveness and extend its application in pharmaceutical product development and assessment.

## **Introduction**

In the pursuit of better medications, the pharmaceutical industry continues to explore new drugs and improve existing drugs. Tablets are one of the most commonly used and stable forms of medication due to their higher patient acceptance, ease of consumption, safety, portability, higher physicochemical and microbiological stability, and efficient production capabilities. Among these, rapidly disintegrating tablets, due to their quick release of contents after consumption and rapid interaction with gastrointestinal fluids in less than 3 min, have gained increased patient favor and popularity [1, 2]. 

Tablets, which are prepared through the compression of one or more active pharmaceutical ingredients (APIs) along with excipients, can vary in effectiveness based on the formulation type. While excipients do not possess medicinal properties, their choice is crucial because of their impact on the final product quality, making it a critical element in tablet formulation design [3–7]. 

The appropriate selection of excipients for direct tablet compression can enhance the quality, stability, and desired performance of pharmaceutical tablets, ultimately contributing to the success and effectiveness of this dosage form. The tablets characteristics are influenced by the physicochemical properties of the active ingredient, formulation factors, and process parameters. These attributes hold special importance in the design of brand name drug formulations, and predicting them given the diversity of influential specifications can be challenging [8, 9].Understanding these connections is crucial in fabricating pharmaceutical products for improved quality and efficacy [10]. 

The development of pharmaceutical formulations involve pre-formulation experiments carried out through trial and error, a method that is time-consuming, costly, labor-intensive and leads to environmental pollution [11]. Consequently, simplification and automation of this process are crucial in the pharmaceutical industry [12]. The key parameter that requires optimization in the development of fast-disintegrating tablets is the tablet’s disintegration time. Initially, these tablets contain a variety of excipients. Parameters such as friability, hardness, and disintegration time are then evaluated to identify the best formulation balance, aiming for minimal disintegration time alongside suitable hardness and friability [13].The ODTs formulation design is essential for reducing disintegration time without compromising the tablet’s quality [14]. 

In the past decade, numerous studies, have been conducted in the field of pre-formulation drug design, with a focus on the automation and learnability of this process through approaches based on machine learning techniques, [15–23], The utilization of these techniques can accelerate and facilitate the development process, formulation optimization, and lead to cost reductions [20].

The utilization of artificial intelligence technology in the pharmaceutical industry has experienced significant growth in recent years. Numerous studies have confirmed the effectiveness of AI in various applications. A considerable number of these studies have demonstrated that deep learning consistently outperforms other machine learning methods in predicting the dissolution or tablets disintegration times, drug solubility in water, and drug discovery and identification [14, 24–28]. However, studies that represent machine learning or deep learning models in pharmaceutical formulations were carried out with constrained data conditions and a restricted dataset. Considering the superior ability of deep learning methodologies over conventional machine learning approaches in handling vast data volumes and empowering analysis, the inclusion of more data is likely to yield improved outcomes. Therefore, this study aimed to develop a prediction model employing advanced machine learning techniques and deep learning, aiming to assist in the pre-formulation drug design process. This enhancement involves reducing the initial stages and iterations to find the best solution, as well as minimizing the consumption of primary drug materials. The created model accurately predicts two important parameters—disintegration time and hardness—in control tests for fast-disintegrating tablets. This is accomplished by integrating additional input data, a dataset resulting from aggregating formulations under various laboratory conditions, enriched with many of records from multiple studies, enhancing its reliability and precision in assessing these crucial tablet properties.

## **Method**

This study represents the second phase of a two-phase project, encompassing dataset creation and model design. In the initial phase, our focus was on compiling a comprehensive dataset of rapidly disintegrating tablet formulations, which involved four key stages: extracting articles from databases, compiling article specifications, extracting formulations from selected articles, and preprocessing and cleaning the data. These stages are elaborated upon in the article [29]. In the second phase, presented in this study, we leveraged the dataset created in the first phase to develop predictive models for estimating disintegration time and tablet hardness. This phase involved three main stages, namely, data preprocessing, data splitting, and development, which centered on the design and evaluation of predictive models using deep learning and machine learning techniques.

## Data description

**Fig. 1:**
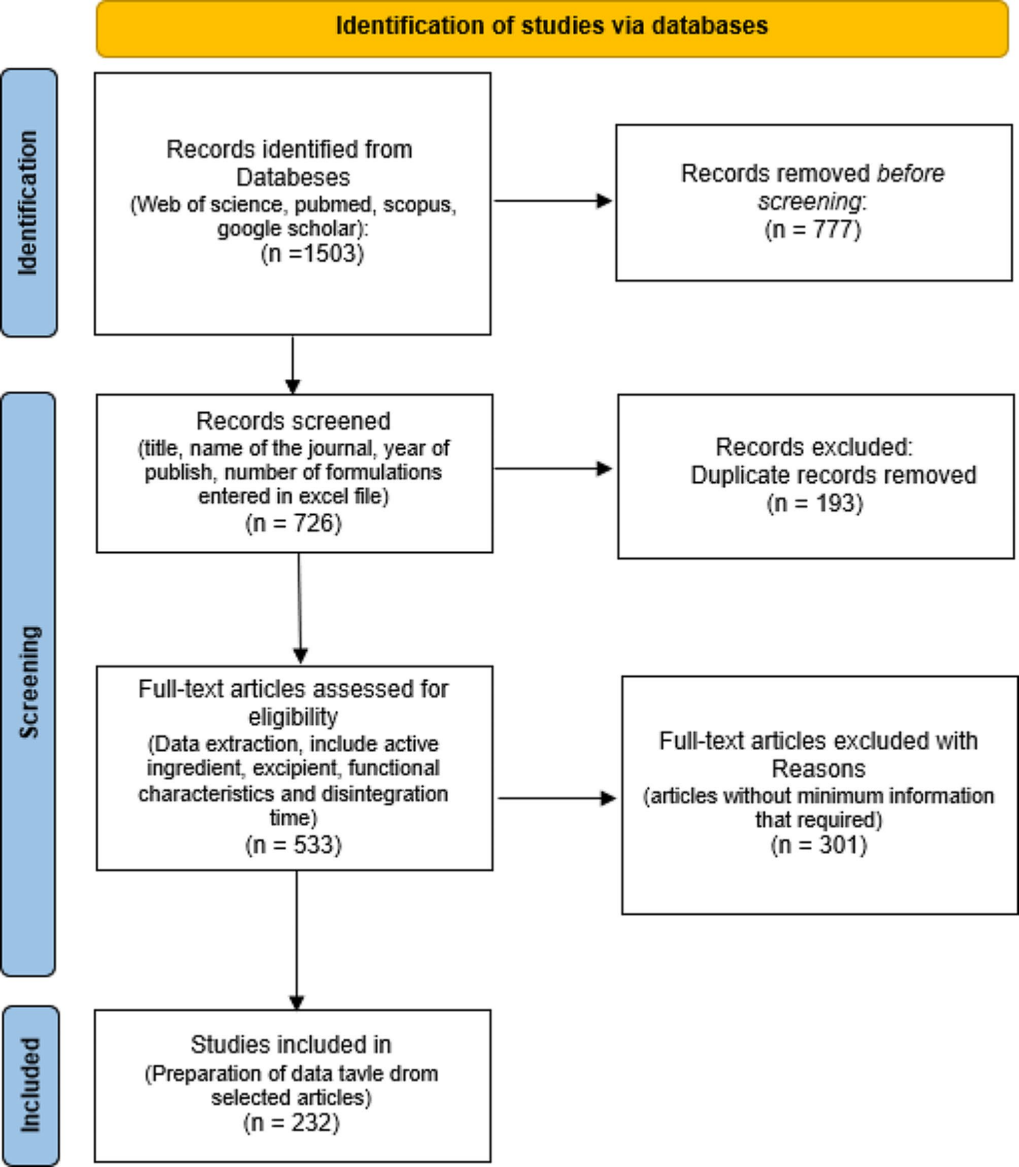
Stages of extracting selected articles [29]

The dataset from the initial phase of the aforementioned project consisted of 1983 formulations, each associated with 78 features. These formulations were obtained from articles retrieved from databases such as PubMed, Web of Science, Scopus, and Google Scholar, spanning from 2010 to 2020. A visual representation of the dataset creation process employed in this research is depicted in Fig. 1 [29]. This research focused on analyzing two characteristics, disintegration time and tablet hardness, as key variables of interest given their significance in the formulation process.

The dataset utilized in this study contains comprehensive information crucial for formulating and assessing pharmaceutical products. Table 1 outlines a summary of this dataset, incorporating different aspects related to the active ingredient, excipients, and structural features. Categorized into seven general dimensions, this dataset presents valuable insights into the formulation process, facilitating a comprehensive understanding of formulation characteristics.

**Table 1:** Summary of dataset dimensions and attributes

Dimension	Attributes
Quality control tests for formulations	Disintegration Time
	Hardness
	Friability
	Water Absorption Ratio
Excipients	Excipient 1 Amount
	Excipient 2 Amount
	…
	Excipient 56 Amount
Characteristics of powdered material composition	Bulk Density
	Tapped Density
	Carr’s Compressibility Index
	Hausner Ratio
	Angle of Repose
Physical attributes of the tablet after blending and compression	Thickness
	Wetting Time
Total tablet weight	Tablet Weight
Amount of active ingredient	Active ingredient weight
Physicochemical properties of the active ingredient	Molecular Weight
	XLogP3-AA
	Hydrogen Bond Donor Count
	Hydrogen Bond Acceptor Count
	Rotational Bond Count
	Topological Surface Area
	Heavy Atom Count
	Complexity
	LogS

## Modeling

The research was structured into three phases. Initially, data preprocessing procedures were implemented (2.1). Subsequently, the study dataset was segmented into three sections: training, validation, and test datasets (2.2). The final phase centered on constructing predictive models (2.3).

### Data preprocessing

At this point, the data preprocessing and cleaning were performed, and in the following, various steps are detailed separately:

The steps involved in standardizing excipient and active ingredient names, as well as structural property values, included normalizing excipient material names (abbreviations and alternate titles), compiling a standardized list of active ingredients (abbreviations, alternative names, converting brand names to generic names), aligning units for structural properties, setting the mean as the midpoint and one-sixth of the range as the standard deviation for values reported within a numerical range. Additionally, the data was cleaned by reformatting it from a multi-row structure to a single record per formulation for better processing, and extreme data values (such as outliers related to disintegration time) were removed from consideration by deleting corresponding records.

### Data splitting

Before the modeling process, the research dataset was split into training, validation, and test datasets for each algorithm. This was accomplished by employing the Repeated Random Train-Validation-Test split method, where random splits were performed multiple times to attain a more reliable performance assessment. Eventually, the optimal splitting percentage was determined for each method. Based on these principles, in the deep learning method, 85% of the dataset was allocated for the training set, 5% for the validation set, and 10% for the test set. In the artificial neural network method, these ratios were 90%, 5%, and 5% respectively. For other machine learning methods, an 80% training set and a 20% test set were considered for the data.

The training dataset was used for model training and learning. The validation dataset was also utilized for fine-tuning the hyperparameters of the algorithms, aimed at enhancing performance and preventing overfitting, particularly in the case of deep learning models and artificial neural networks exclusively. Finally, the test dataset was used to assess the model’s performance in predicting new data.

### Development

Given the prevalence of machine learning and deep learning methods in the field of pharmaceutical technologies and considering the nature of the formulation dataset which includes formulation compositions and process control tests for drug manufacturing (17, 31, 39, 72, 73) (39), in this study, deep learning methods were employed, with the primary focus on the fully connected deep neural network architecture.

In addition to deep neural networks (DNNs), other methods such as artificial neural networks (ANNs), support vector machines (SVMs), general neural network regression (GRNNs), decision trees (DTs), multiple linear regression (MLR), and the random forest (RF) ensemble method, were used for investigation and comparison. This choice was made due to the relevance of these techniques in pharmaceutical research and the potential benefits they offer in extracting insights from complex data.

We conducted hyperparameter optimization to fine-tune the parameters of our models for optimal performance. This involved systematically testing and evaluating various combinations of hyperparameters using techniques such as grid searches and random searches. Specifically, we explored different values for each hyperparameter, including learning rates, batch sizes, activation functions, and regularization techniques. The goal was to identify the set of hyperparameters that maximized the performance metrics of our models, such as accuracy or mean squared error. After extensive experimentation and comparison, we selected the hyperparameters that yielded the best results based on our evaluation criteria.

#### Hyperparameters of the deep learning method

**Fig. 2:**
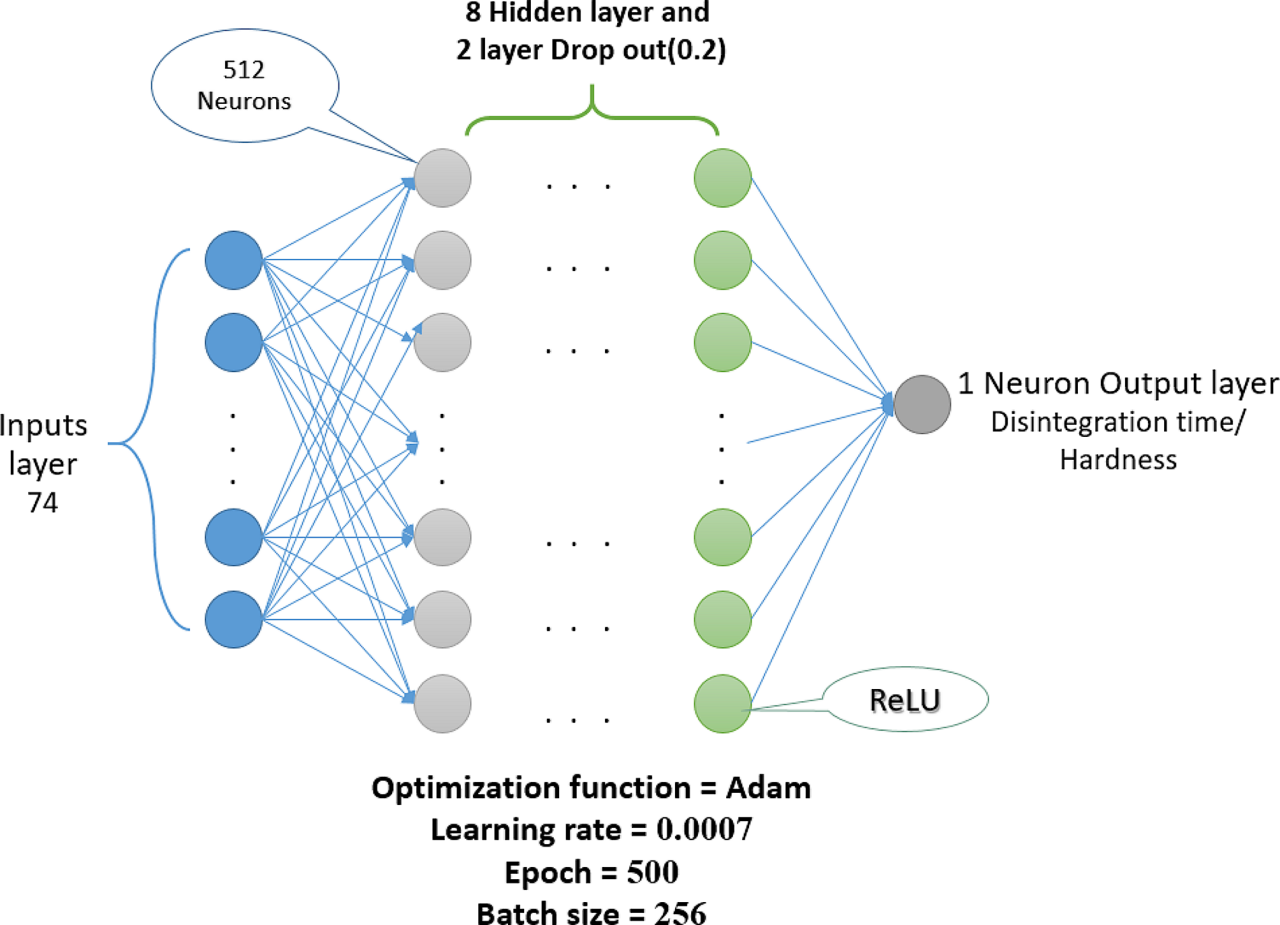
Architecture of the deep neural network

For the prediction of disintegration time, as illustrated in Fig. 2, the deep neural network is structured as a 12-layer network comprising 8 hidden layers, two dropout layers (with a dropout rate of 0.2) to mitigate overfitting, and input and output layers. This architecture was carefully designed to optimize the model’s performance and generalization ability. In each hidden layer, a total of 512 neurons were placed, and the activation function for each of these hidden layers was set as the ‘Rectified Linear Unit (ReLU)’ function. Considering that the model is a regression model and needs to produce a single estimated value in the final layer, no activation function was applied to this layer. During model training, the Adam optimizer was employed with a learning rate of 0.0007 over 500 epochs, while the batch size was set to 256. For predicting hardness, the deep neural network had a similar structure, with one less hidden layer, and the number of neurons in the hidden layers was adjusted to 320. The learning rate was set to 0.001.

#### Hyperparameters of machine learning methods

Regression models were developed using various machine learning methods for comparison with DNNs, including ANNs, SVMs, GRNNs, DTs, MLR, and RF ensemble method. These models were trained using the scikit-learn package. The artificial neural network was designed with an input layer, a hidden layer with 256 neurons, and an output layer. The ReLU served as the activation function in the hidden layers, and the Adam optimizer was used with a learning rate of 0.001. For SVM, a linear kernel was employed, whereas an RBF kernel was used for GRNN. In the case of RF, the number of decision trees in the forest (n_estimators) was set to 30.

## Evaluation

As selecting a model that exhibits desirable accuracy and precision is crucial in the process of designing and developing predictive models, in this study, various performance measurement methods (such as Mean Squared Error, Coefficient of Determination, and Mean Absolute Error) were considered to assess the predictive accuracy and compare different models. The formulas for each of these metrics are as shown below:

- Root Mean Squared Error (RMSE).

$$ RMSE=\sqrt{\frac{1}{N} \sum _{j=1}^{N}{({target}_{j}- {output}_{j})}^{2}}$$

The RMSE measures the average deviation between predicted values (output) and actual values (target). It is calculated by taking the square root of the average of the squared differences between each predicted and actual value.

- Coefficient of determination (R^2^).

$$ {R^2} = \frac{{\sum\nolimits_{i = 1}^n {{{(outpu{t_i} - \mathop {target}\limits^ - )}^2}} }}{{\sum\nolimits_{i = 1}^n {{{(targe{t_i} - \mathop {target}\limits^ - )}^2}} }}$$

The R^2^ value, also known as the coefficient of determination, indicates the proportion of the variance in the dependent variable(s) that is predictable from the independent variables in a regression model. It ranges from 0 to 1, where 1 indicates a perfect fit.

- Mean Absolute Error (MAE).

$$ MAE= \frac{1}{N}{\sum }_{J=1}^{N}\left|{target}_{j}-{output}_{j}\right|$$

The MAE represents the average absolute difference between the predicted (output) and actual (target) values. It provides a measure of the model’s accuracy in predicting the target variable. To assess the accuracy of the model within three different tolerance levels of the predicted values, the following formulas were applied. The rationale behind this calculation is as follows: if the discrepancy between the predicted value and the true value is less than 10% (15%, 20%) of the true value, it is considered a correct prediction. To assess the model’s accuracy within three different percent tolerances of the predicted values, the following formulas were employed. The underlying principle of this calculation is as follows: if the difference between the predicted value and the actual value is less than 10% (15%, 20%) of the actual value, it is deemed a correct prediction.

1 $$ Accurac{y_{tolerance = 10\% }} = \frac{{Number\,(\left| {{Y_i} - {{\hat Y}_i}} \right| \le 0.1 * \hat Y)}}{{All\,predictions}}$$

2 $$ Accurac{y_{tolerance = 15\% }} = \frac{{Number\,(\left| {{Y_i} - {{\hat Y}_i}} \right| \le 0.15 * \hat Y)}}{{All\,predictions}}$$

3 $$ Accurac{y_{tolerance = 20\% }} = \frac{{Number\,(\left| {{Y_i} - {{\hat Y}_i}} \right| \le 0.2 * \hat Y)}}{{All\,predictions}}$$

To implement the predictive models, Python version 3.6.3 was utilized, along with the PyCharm 2020.3 programming environment.

## Result

The predicted results of various models for the “disintegration time” response variable can be found in Table 1, while those for the “hardness” of tablets are detailed in Table 2.

**Table 2:** Disintegration time variable

Method	Split Train . Validation :test	MAE		RMSE		R^2^		Accuracy tolerance = 10%		Accuracy tolerance = 15%		Accuracy tolerance = 20%	
		Train	Test	Train	Test	Train	Test	Train	Test	Train	Test	Train	Test
DNN	85.5:10	5.54	10.65	12.34	23.96	0.92	0.77	0.63	0.44	0.83	0.60	0.92	0.73
ANN	90.5:5	11.89	12.00	34.64	23.52	0.43	0.52	0.62	0.47	0.70	0.54	0.77	0.67
GRNN	90:10	0.47	12.88	5.25	31.90	0.98	0.35	0.98	0.34	0.98	0.54	0.99	0.64
DT	80:20	0.009	16.38	0.26	34.72	0.99	0.36	0.99	0.29	0.99	0.40	1.0	0.50
MLR	80:20	22.18	23.27	37.19	36.27	0.31	0.41	0.18	0.24	0.24	0.24	0.31	0.30
SVR	80:20	20.29	19.68	40.32	34.50	0.25	0.24	0.24	0.22	0.32	0.29	0.39	0.35
RF	80:20	4.72	11.99	10.15	23.33	0.95	0.70	0.68	0.34	0.80	0.46	0.86	0.56

Table 2 presents the predictive performance metrics of various models for the “disintegration time” variable. The MAE, RMSE, R^2^, and accuracy metrics are provided for each model, along with the train-test split ratio.

MAE metric measures the average absolute difference between the predicted and actual disintegration times. Lower values indicate better predictive accuracy.

The RMSE represents the square root of the average squared difference between the predicted and actual values. It provides a measure of the overall error of the model predictions, with lower values indicating better performance.

R^2^ indicates the proportion of the variance in the response variable that is explained by the model. Higher values closer to 1 suggest better fit and predictive capability.

Accuracy metrics are provided for different tolerance levels, indicating the percentage of predictions that fall within a certain percentage tolerance of the actual values. the details of calculation accuracy metric are introduced in the Methods section.

The results demonstrate the performance of each model in accurately predicting the disintegration time of tablets, with the DNN model achieving notably high accuracy across various metrics compared to other methods.

**Table 3:** Hardness tablet response variable

Method	Split Train . Validation :test	MAE		RMSE		R^2^		Accuracy tolerance = 10%		Accuracy tolerance = 15%		Accuracy tolerance = 20%	
		Train	Test	Train	Test	Train	Test	Train	Test	Train	Test	Train	Test
DNN	90.5:5	0.13	**0.20**	0.35	**0.28**	0.92	**0.91**	0.92	**0.85**	0.96	**0.98**	0.98	**0.99**
ANN	90.5:5	0.20	0.24	0.65	0.38	0.74	0.82	0.87	0.79	0.93	0.89	0.95	0.95
GRNN	90:10	0.009	0.35	0.05	0.85	0.99	0.20	0.99	0.74	0.99	0.83	0.99	0.89
DT	80:20	0.0005	0.31	0.01	0.52	0.99	0.87	1.0	0.67	1.0	0.80	1.0	0.88
MLR	80:20	0.65	1.72	1.07	19.47	0.27	0.23	0.39	0.36	0.53	0.50	0.65	0.62
SVR	80:20	0.60	0.65	1.08	1.46	0.20	0.10	0.47	0.45	0.60	0.57	0.70	0.68
RF	80:20	0.11	0.32	0.23	0.85	0.96	0.68	0.96	0.73	0.98	0.87	0.99	0.92

Table 3 presents the predictive performance metrics of various models for the “hardness” tablet response variable, similar to Table 2.

Lower MAE values indicate better predictive accuracy, representing the average absolute difference between the predicted and actual hardness values.

Lower RMSE values indicate better overall predictive accuracy, as measured by the square root of the average squared difference between the predicted and actual hardness values.

Higher R^2^ values indicate a better fit of the model to the data, explaining a larger proportion of the variance in hardness values.

Similar to Table 1, accuracy metrics are provided for different tolerance levels, indicating the percentage of predictions within a certain percentage tolerance of the actual hardness values.

The results highlight the superior performance of the deep neural network (DNN) model in accurately predicting tablet hardness compared to other methods, as evidenced by higher accuracy metrics across various tolerance levels.

**Fig. 3:**
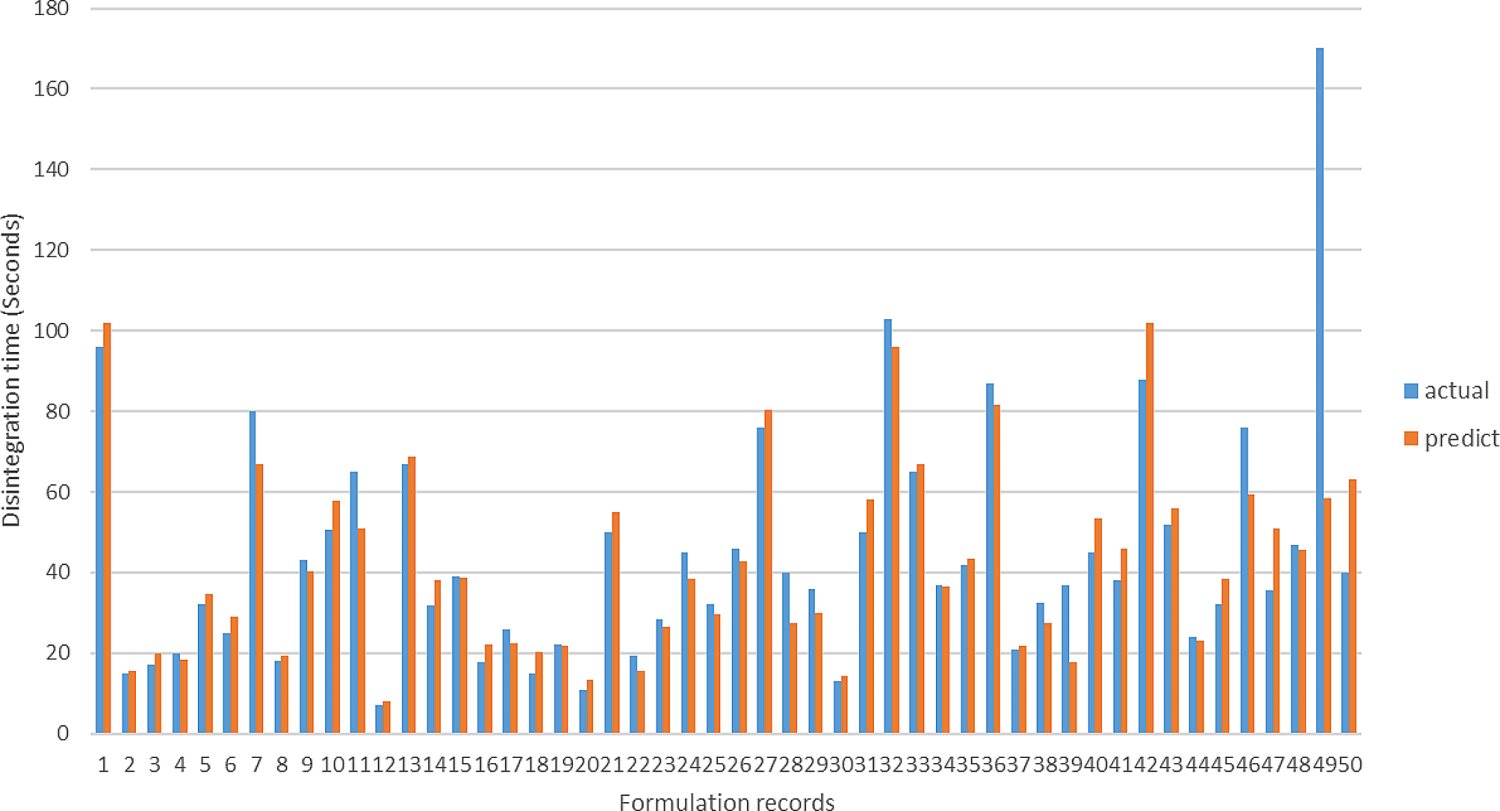
Predicted disintegration time values vs actual values - deep learning method

**Fig. 4:**
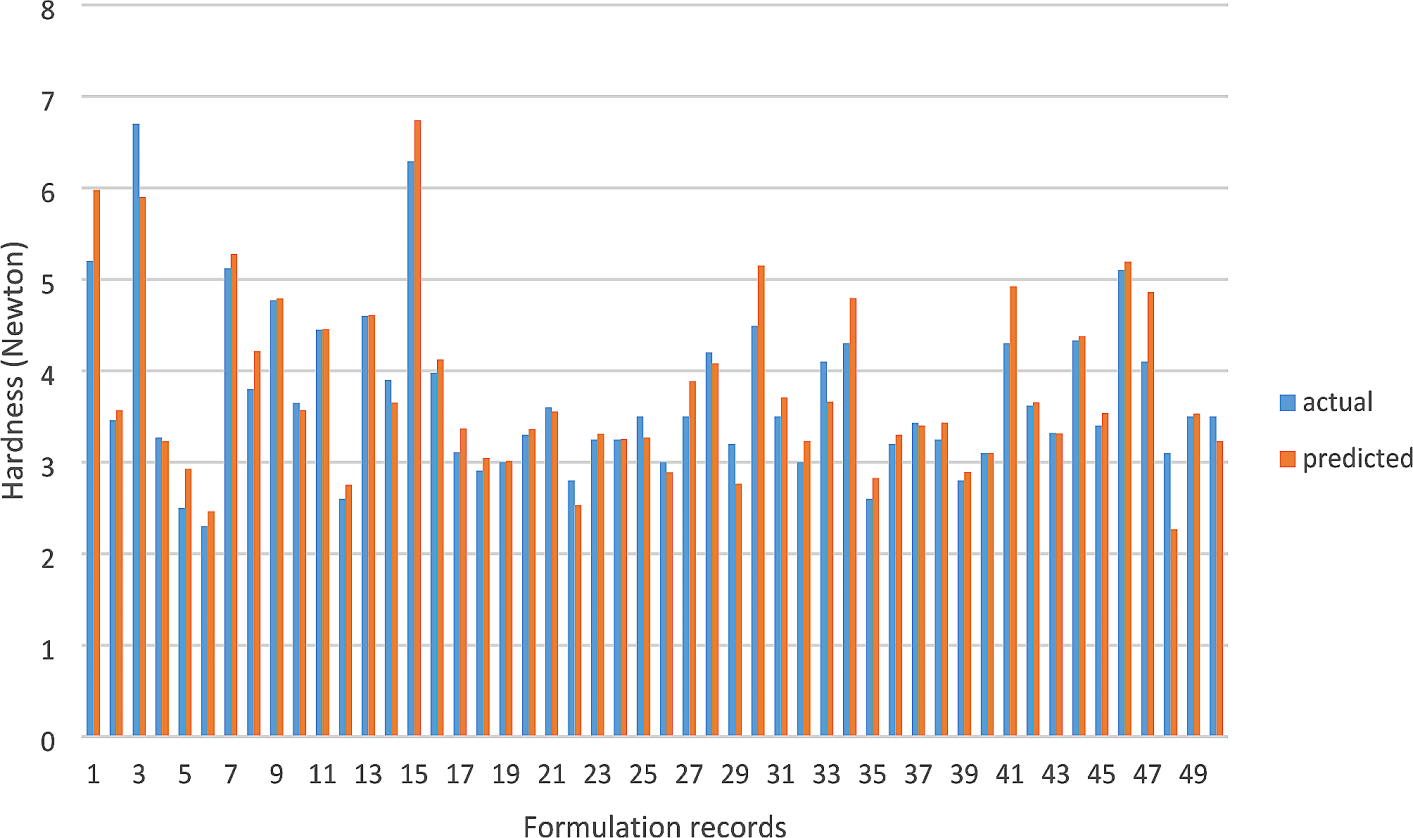
Predicted hardness values vs. actual values - deep learning method

In Figs. 3 and 4, the predicted values generated by the deep neural network predictive model are shown alongside the actual disintegration time and hardness values of the tablets within the dataset. To offer additional explanation regarding the comparison of predictions, Table 4 illustrates ten sample data points, showing the actual disintegration time and hardness, as well as the model’s corresponding predictions. This tabular format provides a thorough analysis of the model’s predictive precision at the level of each individual sample.

**Table 4:** Sample records of predicted values vs actual values

Disintegration time (Second)		Hardness (Newton)	
Actual	**Prediction**	**Actual**	**Prediction**
96	101.95	5.2	5.97
15	15.48	3.46	3.57
17	20.07	6.7	5.90
20	18.42	3.27	3.23
32	34.76	2.5	2.93
25	29.09	2.3	2.46
80	66.82	5.12	5.28
18.15	19.22	3.8	4.21
43	40.15	4.77	4.79
50.5	57.92	3.65	3.57

## Discussion

The main objective of this study is to improve the optimization of pharmaceutical formulations by utilizing artificial intelligence, particularly deep learning techniques. The approach involved creating predictive models to automate the optimization process, ultimately reducing the number of iterations and material usage in experimental tablet manufacturing and testing.

The automation of this process is realized through the development of a predictive model. To identify the most effective predictive model, a range of methods including deep learning techniques, artificial neural networks, support vector machines, general neural network regression, decision trees, multiple linear regression, and the ensemble method of random forest were employed. These methodologies were subsequently compared using assessment metrics such as the Mean Squared Error, Coefficient of Determination, and Mean Absolute Error.

Given the complexity of tablet contents and their manufacturing process, predicting variables such as disintegration time and tablet hardness using conventional and outdated methods is not feasible. These variables are correlated with physical characteristics such as particle size, shape, and tablet thickness, as well as formulation components (excipients and active ingredients) [30]. 

Prediction models were designed and implemented on the training dataset, and subsequently, these models were evaluated using the test dataset.

The accuracy (tolerance of 20%) of the machine learning techniques (ANN, GRNN, DT, MLR, SMR, RF) for predicting the disintegration time variable were 67%, 64%, 50%, 30%, 35%, and 56%, respectively. The corresponding R^2^ values for the same methods were 0.52, 0.35, 0.36, 0.41, 0.24, and 0.70.

Similarly, for the tablet hardness variable, the accuracy rates of the machine learning techniques (ANN, GRNN, DT, MLR, SMR, RF) were 95%, 89%, 88%, 62%, 68%, and 92%, respectively. The corresponding R^2^ values for the same methods were 0.82, 0.20, 0.87, 0.23, 0.10, and 0.68.

Additionally, the accuracy of the deep learning technique for predicting the disintegration time variable (20% tolerance) was 73%. The corresponding R^2^ for this variable was 77%.

For the tablet hardness response variable, the accuracy rate achieved using the deep learning technique was 99%, with an R^2^ value of 91%. These outcomes highlight the effectiveness of the deep learning approach in predicting the specified response variables and the correlation captured by the R^2^ metric.

By comparing the results obtained from various models on the dataset of this study, it becomes evident that the deep learning method exhibited superior performance.

The tablet hardness response variable exhibited superior performance compared to the disintegration time. This variation in performance can be attributed to the inherent characteristics of the variable, which is influenced by a greater amount of interdependencies.

**Fig. 5:**
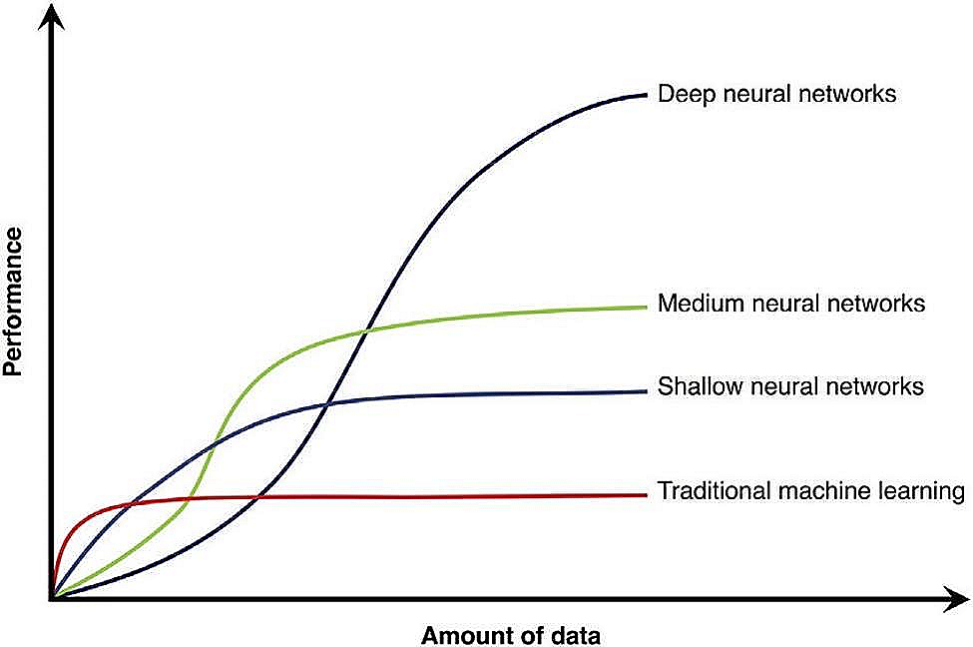
Comparison of different methods with data volume increases [31]

As shown in Fig. 5, considering the results and characteristics of deep learning methods, it can be concluded that increasing the amount of data will improve the model’s performance. In contrast, conventional machine learning models do not show any performance improvement when reaching a specific threshold, even with increased data.

As indicated in a prior investigation [10], the impact of active ingredient properties on tablet disintegration time is more intricate than that on tablet hardness. This complexity poses a more challenging prediction task. To address this challenge and enhance predictive accuracy, a larger dataset containing a diverse range of active ingredients will be necessary. The findings of the current research support and confirm this assertion.

In a similar study [14], which was conducted for rapidly disintegrating tablets, the study dataset consisted of only 145 formulations. Deep learning methods and neural networks were implemented for the data. the authors of the article set the response variable range (disintegration time) in the study dataset from zero to 100 as the basis. They defined 10% of this range as the tolerance threshold for their evaluation. If the difference between the predicted and actual values was less than 10 s, it was considered a correct estimate. Using this criterion, deep learning achieved an accuracy of 80% on the test data.

Following discussions with specialists in the field, it was recognized that this approach (constant value) might pose limitations when dealing with data featuring short disintegration times. This could introduce bias and render the method unsuitable for model fitting and evaluation. Consequently, in the present study, the tolerance percentage was employed as the evaluation metric for the assessment and comparison of the predicted responses. This approach is deemed to be a more appropriate metric for this scenario. (Eq. 1–3)

In a study conducted in 2016 [30], the prediction of response variables for disintegration time and tablet hardness was carried out using non-destructive methods of ultrasound waves and machine learning. The aim of this research was to establish a practical and expandable framework for the rational design and evaluation of various pharmaceutical products in the pharmaceutical sciences. They focused solely on variables related to physical and mechanical characteristics, as well as the process parameters of tablet production for modeling. Factors that were formulation-dependent and had limited generalizability, such as excipients used in the formulation, were neglected. However, in the present study, in addition to physical characteristics, the attributes of the powdered mixture of excipients and active ingredients, the physicochemical attributes of the active substance, and the individual quantities of excipients were incorporated into the model. This broadened approach was taken to capture a more comprehensive representation of the formulation and its constituents.

Despite the valuable insights gained from this study, it is important to acknowledge its limitations. These limitations delineate the scope within which our findings can be interpreted and emphasize areas that warrant further investigation. Due to insufficient data for each active ingredient, especially in formulations with a disintegration time exceeding 180 s, there was a noticeable difference between the estimated time and the actual time. Therefore, it can be concluded that the model will perform better when an adequate number of instances are available for each active ingredient.

Furthermore, another limitation of this study was the use of data from various studies, which may have differences in terms of the devices used to measure the response variable, the rotation speed of the blender, the pressure of the tablet press machine, and the method of mixing the ingredients. Due to the high diversity of these factors and the absence of references to the types of devices used in some articles, it was not possible to standardize them. These factors could have an impact on the response, but they were not considered in this study.

In previous and similar studies [10, 14, 28, 30, 32] all the data used in the models were extracted in limited quantities and from a single laboratory with consistent settings. However, in this study, a comprehensive dataset of formulations was used, and the data were extracted from various sources [29].Therefore, considering the diversity of the data and the wide range of device settings used, this data diversity can be considered a prominent feature of this research. Achieving an accuracy of 71% in predicting tablet disintegration time under the current study conditions is desirable. Hence, it can be claimed that the models presented in this study can provide acceptable predictions of response variables for new data with different settings. This is a notable strength of the current study.

Some variables, such as tablet hardness, are measured after the final tablet production, and therefore, their values are not available during the formulation design phase. However, considering the significant impact of this variable on tablet disintegration time, as indicated in study [30], in future research on the dataset [29], it may be beneficial to predict this variable. Subsequently, using the predicted value as one of the predictive variables in the tablet formulation prediction model can potentially enhance the model’s performance and should be explored to improve model accuracy and reliability.

In future studies, tablet disintegration time could be considered a qualitative variable and classification machine learning methods and deep learning techniques could be applied. This approach can offer various applications compared to the quantitative approach in industry. By doing so, it may provide valuable insights and potentially improve the prediction and control of tablet disintegration behavior, leading to more efficient and versatile applications in pharmaceutical and related industries.

To comprehensively assess the models created for future use as practical tools, given the diverse nature of excipients and the sparsity of the databases in the columns related to them, improving the models can involve creating another dataset by aggregating excipients based on their performance in tablet formulations. Creating a supplementary dataset that focuses on excipient performance can be beneficial. This dataset can provide a more detailed understanding of how different excipients influence tablet properties and disintegration behavior. By expanding the dataset with more excipient-related features and their effects on tablet characteristics, the model’s predictive capabilities can be enhanced, making it a more valuable tool for future applications, especially in the pharmaceutical industry.

Furthermore, deep learning techniques have been used for different tasks, including sequence feature analysis [33] and clustering [34], and they also offer significant potential for optimizing pharmaceutical formulations in the future.

The findings of this study underscore the importance of data diversity and model sophistication in achieving accurate predictions, ultimately contributing to more efficient pharmaceutical manufacturing processes.

## Conclusion

In conclusion, our research presents a groundbreaking approach to pharmaceutical formulation by harnessing the power of machine learning and deep learning techniques. We addressed the challenges in pre-formulation drug design, including the time-consuming and costly trial-and-error process. Through the analysis of a comprehensive dataset comprising formulation details and physicochemical attributes, we successfully predicted two critical control test parameters: disintegration time and tablet hardness.

Our study demonstrated that deep learning, particularly a 12-layer deep neural network, outperformed other methods, achieving remarkable accuracy in predicting these complex pharmaceutical variables. This approach offers the potential to streamline the drug formulation process, reducing the need for extensive iterations and saving valuable consumable materials.

As the pharmaceutical industry continues to evolve, the utilization of deep learning and machine learning techniques holds promise for optimizing pharmaceutical product development. Future work should focus on expanding the dataset, improving model performance, and exploring broader applications in pharmaceutical design and evaluation. Ultimately, our research contributes to the advancement of pharmaceutical science and innovation.

## Data Availability

The datasets analyzed during the current study, as detailed in reference (29), is readily available for open access. Interested parties can freely explore and examine the specifics of pre-formulation tests on fast disintegrating tablets (FDTs) under 10.7910/DVN/TUSJYB (35) through the provided source, ensuring transparency and facilitating further analysis or validation of the findings.

## Abbreviations

FDT: fast disintegrating tablets
API: Active Pharmaceutical Ingredient
DNN: deep neural networks
ANN: artificial neural networks
SVM: support vector machines
GRNN: general neural network regression
DT: decision trees
MLR: multiple linear regression
RF: random forest
ODTs: orally disintegration tablets
ReLU: Rectified Linear Unit
RMSE: Root Mean Squared Error
MAE: Mean Absolute Error
